# High-intensity-focused ultrasound in the treatment of primary prostate cancer: the first UK series

**DOI:** 10.1038/sj.bjc.6605116

**Published:** 2009-06-09

**Authors:** H U Ahmed, E Zacharakis, T Dudderidge, J N Armitage, R Scott, J Calleary, R Illing, A Kirkham, A Freeman, C Ogden, C Allen, M Emberton

**Affiliations:** 1 Division of Surgery and Interventional Sciences, University College London, 67 Riding House Street, London W1P 7PN, UK; 2 Clinical Effectiveness Unit, The Royal College of Surgeons of England, 35-43 Lincoln Inn Fields, London, UK; 3 The Academic Department of Radiology, University College London Hospitals NHS Trust, 235 Euston Road, London NW1 2BU, UK; 4 The Department of Histopathology, University College London Hospitals NHS Trust, London, UK; 5 The Royal Marsden Hospital, London, UK

**Keywords:** HIFU, high-intensity-focused ultrasound, transrectal, prostate cancer, Sonablate500

## Abstract

**Background:**

The use of minimally invasive ablative therapies in localised prostate cancer offer potential for a middle ground between active surveillance and radical therapy.

**Methods:**

An analysis of men with organ-confined prostate cancer treated with transrectal whole-gland HIFU (Sonablate 500) between 1 February 2005 and 15 May 2007 was carried out in two centres. Outcome data (side-effects using validated patient questionnaires, biochemical, histology) were evaluated.

**Results:**

A total of 172 men were treated under general anaesthetic as day-case procedures with 78% discharged a mean 5 h after treatment. Mean follow-up was 346 days (range 135–759 days). Urethral stricture was significantly lower in those with suprapubic catheter compared with urethral catheters (19.4 *vs* 40.4%, *P*=0.005). Antibiotics were given to 23.8% of patients for presumed urinary tract infection and the rate of epididymitis was 7.6%. Potency was maintained in 70% by 12 months, whereas mild stress urinary incontinence (no pads) was reported in 7.0% (12 out of 172) with a further 0.6% (1 out of 172) requiring pads. There was no rectal toxicity and no recto-urethral fistulae. In all, 78.3% achieved a PSA nadir ⩽0.5 *μ*g ml^−1^ at 12 months, with 57.8% achieving ⩽0.2 *μ*g ml^−1^. Then, 8 out of 13 were retreated with HIFU, one had salvage external beam radiotherapy and four chose active surveillance for small-volume low-risk disease. Overall, there was no evidence of disease (PSA <0.5 *μ*g ml^−1^ or negative biopsy if nadir not achieved) after one HIFU session in 92.4% (159 out of 172) of patients.

**Conclusion:**

HIFU is a minimally invasive, day-case ablative technique that can achieve good biochemical outcomes in the short term with minimal urinary incontinence and acceptable levels of erectile dysfunction. Long-term outcome needs further evaluation and the inception of an international registry for cases treated using HIFU will significantly aid this health technology assessment.

Men diagnosed with localised prostate cancer have to choose between active surveillance or radical therapy in the form of prostatectomy or radiotherapy (Bill-Axelson *et al*, 2005). Choosing between these extremes of care is not easy, as radical treatments come with a significant risk of morbidity (Zeliadt *et al*, 2006; Wilt *et al*, 2008). In contrast, surveillance strategies confer minimal treatment-related harms, but carry potential psychological burden and delayed radical therapy in up to one-third of the patients as a result of disease progression (van As and Parker, 2007). Ablative therapies have the potential to offer a middle ground between these extremes. They may confer different patterns of toxicity compared with standard radical therapies and at the same time may offer greater freedom from progression than that from surveillance strategies. A number of platforms – cryosurgery, high-intensity-focused ultrasound (HIFU), radiofrequency ablation and photodynamic therapy – are in various stages of evolution, evaluation and diffusion into clinical practise.

In this paper, we report our early experience with HIFU. HIFU was evaluated following UK's National Institute of Clinical Excellence (NICE) guidance issued in 2005 stating they ‘… support the use of this procedure for the treatment of prostate cancer’ but with the caveat that ‘… audit should address clinical outcomes, long-term survival and indications for treatment’ (NICE Guideline on High-Intensity Focused Ultrasound Therapy for Prostate Cancer, 2005). In line with this request, we present our data on the use of HIFU in treating men with prostate cancer. We have adhered to Strengthening the Reporting of Observational Studies in Epidemiology (STROBE) recommendations which are appropriate in this setting (Vandenbroucke *et al*, 2007).

## Background

HIFU relies on the physical properties of ultrasound, which allow it to be brought into a tight focus either using an acoustic lens, bowl-shaped transducer or electronic-phased array. When the energy density at the focus is sufficiently high, tissue damage occurs through coagulative necrosis. The acoustic focal volume of a single exposure is typically 10 mm long by 1–2 mm wide, so larger ablative volumes are created by placing overlapping lesions next to each other. There are two commercially available transrectal devices, the Ablatherm (Edap-Technomed, Lyon, France) and Sonablate 500 (Focus Surgery, Indianapolis, IN, USA). The Sonablate 500 system's treatment planning, execution and monitoring are controlled using an interface which allows the surgeon to target the area of treatment, adjust the focal length of the transducer and alter the power delivered to each focal zone (Illing *et al*, 2006).

## Methods

Men who did not wish to, or were unable to undergo either surgery or radiotherapy for treatment of prostate cancer, and were either not suitable or had rejected surveillance were offered HIFU. These men were treated in two centres (Princess Grace Hospital, London and University College London Hospital NHS Foundation Trust), during the period 1 February 2005 to 15 May 2007, using the Sonablate 500 device. Each man was counselled fully as to his treatment options and specifically told that HIFU was not standard care and that outcomes were limited to short- or medium-term follow-up from case series conducted in France, Germany or Japan. In addition, they were made aware of the reported frequency of side-effects. Men with specific contraindications to HIFU were excluded. These were a prostate volume greater than 40 ml (or a maximum anterior–posterior height greater than 40 mm), any focus of calcification (>10 mm in maximum diameter), significant anorectal disease preventing probe insertion (e.g., previous haemorrhoidectomy) or carrying increased risk of fistulae (e.g., Crohn’s disease). To achieve these size restrictions, some were pretreated with low-dose antiandrogen monotherapy for 3 months to reduce the gland size.

During the period in question some of our practises altered. At the outset, all men were fitted with a urethral catheter for 7–10 days, but after consultation held in international user group meetings it was agreed that suprapubic catheters might result in earlier voiding, fewer urinary infections and lower rates of stricture. As most were tertiary referrals and lived some distance away, all men were taught clean intermittent self-catheterisation, so that they were able to self-manage poor flow or pending retention of urine due to passage of debris. Follow-up after treatment mirrored the regimen used after standard radical therapies: serum PSA measurements at 6 weeks, and then every 3 months for the first year, and every 6 months for subsequent years of follow-up. Patients at one of the centres completed pre- and post-treatment validated questionnaires (International Prostate Symptom Score [IPSS], International Index of Erectile Function-15 (IIEF-15)). Incontinence data were collected from patient reported outcomes on leakage and pad usage. The IIEF-15 data was analysed in two ways. First, we selected a question that is accepted as a good indicator of erectile function (Burnett *et al*, 2007), ‘When you had erections with sexual stimulation, how often were your erections hard enough for penetration?’. We defined potency as scoring either 2 and above or 3 and above on a scale of 0–5: (0) No sexual activity, (1) Almost never/never, (2) A few times (much less than half the time), (3) Sometimes (about half the time), (4) Most times (much more than half the time), (5) Almost always/always. The cohort of 49 completing 12 months of questionnaires with preoperative data were included in the analysis as it provided the most reasonable balance between absolute numbers and duration of follow up. The second form of analysis for erectile function involved a repeated measures analysis comparing mean IIEF-15 score obtained before therapy to that measured at key intervals on follow-up.

Patients not achieving a PSA nadir of ⩽0.5 ng ml^−1^ or those with PSA <0.5 ng ml^−1^ and two consecutive increases in PSA were advised to have a transrectal prostate biopsy. Patients were stratified using 1998 D’Amico risk categories (D’Amico *et al*, 1998).

## Results

### Baseline data

In all, 172 treated with the Sonablate 500 HIFU device for primary prostate adenocarcinoma (⩽T3bN0M0) were identified. None had pre-HIFU transurethral resection of prostate. The mean age was 64.1 years (±s.d. 8.3) with a mean follow-up of 346 days (±s.d. 237, range 135–759). Patients were risk stratified in the 136 men in which this was possible. Here, 27.8% (38 out of 136), 37.5% (51 out of 136) and 34.6% (47 out of 136) were in the low-, intermediate- and high-risk categories. Thirty six patients did not have complete data for risk stratification but for completeness their data was also analysed (Table 1). Thirteen patients were lost to follow-up. However, there was no significant difference in the baseline PSA (10.0 *vs* 8.4, ANOVA *P*=0.522), Gleason score (mean 6.3 *vs* 6.5, ANOVA *P*=0.449) and T-stage (ANOVA *P*=0.666). Around 29% (50 out of 172) of the patients were on 3 months bicalutamide (50 mg once daily) and 5-α reductase inhibitors (dutasteride or finasteride) before HIFU to reduce gland size. Hormones were stopped on the day of treatment. All were treated under general anaesthetic in a single session. Median hospital stay was 5 h (range 3–24 hours) and reasons for overnight stay (22%) were haematuria (no transfusion), co-morbidity (not fit for day-case surgery), lack of home care and side-effects from general anaesthesia.

### Morbidity

Mean catheterisation time was 13.9 days (±s.d. 8.3). In all, 30.2% of men required interventions for either a stricture or necrotic tissue within the prostate cavity. Mode of catheterisation appeared to make a difference to the likelihood of experiencing either a stricture or having retained necrotic tissue. Of those with a suprapubic catheter developed this problem compared to 40.4% in those with a urethral catheter (*P*=0.005, *χ*^2^ test). Strictures or retained necrotic tissue were treated in the following manner: local anaesthetic dilatation, 15.7% (27 out of 52); general anaesthetic dilatation and/or resection, 18.0% (31 out of 52); bladder neck incision, 10.5% (18 out of 52) (Table 2). Any degree of urine leakage was reported regardless as to whether it required pads or a change in underwear or not. Grade 1 stress urinary incontinence (no pads) occurred in 7.6% (13 out of 172) of the patients with 0.6% (1 out of 172) of the patients suffering grade 3 stress urinary incontinence requiring an artificial urinary sphincter. None of the men required security pads. IPSS score significantly deteriorated at 3 and 6 months but returned to baseline at 9 and 12 months. Scores at baseline, 3, 6, 9, and 12 months were 6.7 (±s.d. 5.8), 12.0 (±8.6, *P*<0.01), 9.2 (±8.0, *P*=0.02), 7.9 (±7.5, *P*=0.32), and 7.8 (±s.d. 7.3, *P*=0.42) (*P*-values quoted are comparative to baseline). A degree of dysuria was noted in all men for a week following treatment in those fitted with a suprapubic catheter or following catheter removal in those fitted with a urethral catheter. Around 23.8% of patients received antibiotics at some point during the period of follow-up for a presumed urinary tract infection. In all, 17.9% (12 out of 67) were prescribed antibiotics in the group with a suprapubic catheter compared to 24.4% (23 out of 94) of those with a urethral catheter. Epididymitis occurred in 7.6% (13 out of 172) of patients. Eight of these had a urethral catheter, although the overall percentage with epididymitis in the group with a urethral catheter was 8.5% (8 out of 94) and 7.5% (5 out of 67) in the group with a suprapubic catheter.

About 94 men completed the IIEF-15 questionnaire preoperatively. All were offered phosphodiesterase type 5 inhibitors (either sildenafil or tadalafil). It was not possible to determine from the case notes the number using medication to assist in getting or maintaining erections. The number completing post-treatment questionnaires at 3, 6, 9, and 12 months after treatment were 92, 77, 51, and 34, respectively. 24 out of 51 were deemed potent preoperatively using question 2 threshold of 2 and above. At 3, 6, 9 and 12 months post-HIFU, 37.5% (9 out of 24), 45.8% (11 out of 24), 58.3% (14 out of 24) and 66.7% (16 out of 24) were potent, respectively. When using a threshold value of 3 or more, 12 out of 51 were deemed potent prior to HIFU and 41.7% (5 out of 12), 58.3% (7 out of 12), 66.7% (8 out of 12) and 66.7% (8 out of 12) were potent at 3, 6, 9, and 12 months, respectively. In the second analysis, mean IIEF-15 scores at baseline and then at 3, 6, 9, and 12 months was 33.8, 18.1, 39.1, 23.9, and 28.1, respectively. A *t*-test comparison showed a statistically significant drop in IIEF-15 score at 3 months compared with baseline (*P*<0.01), but no significant difference at other times compared with baseline (Table 2).

### Cancer control outcomes

**Figure 1:**
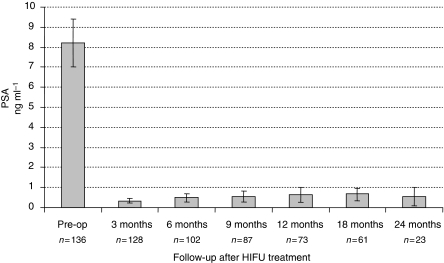
PSA outcome for Localised Prostate Cancer Treated with HIFU.

**Figure 2:**
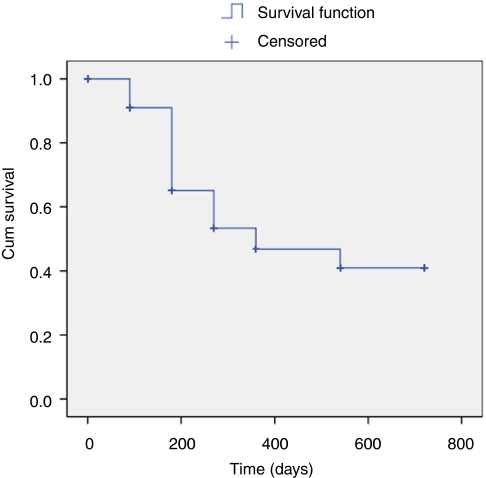
Kaplan Meier curve demonstrating Biochemical Progression Free Survival (PSA </= 0.2 ng/ml) following whole-gland HIFU.

**Figure 3:**
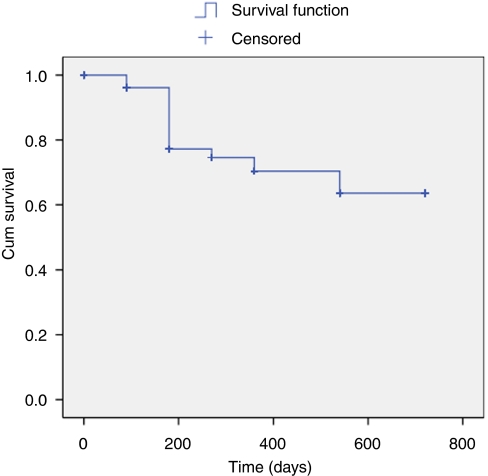
Kaplan Meier curve demonstrating Biochemical Progression Free Survival (PSA </= 0.5 ng/ml) following whole-gland HIFU.

**Figure 4:**
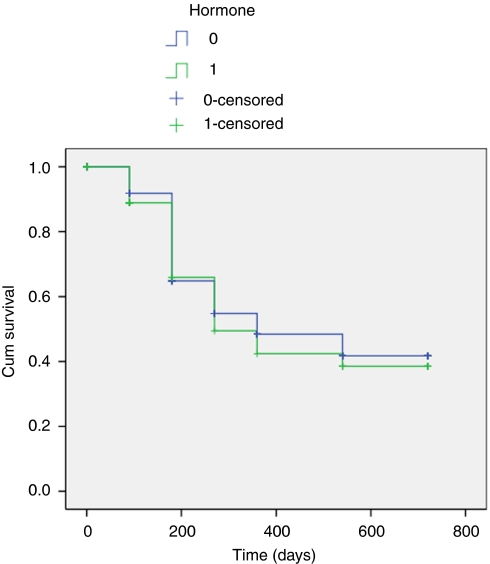
Kaplan Meier curve demonstrating the effect of hormone used as cytoreduction prior to HIFU (PSA </=0.2 ng/ml).

**Figure 5:**
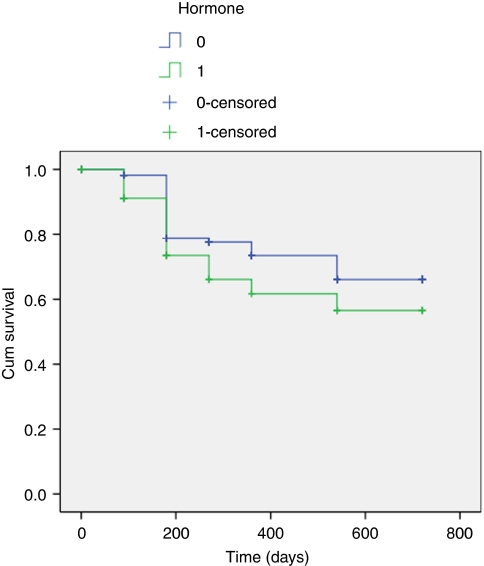
Kaplan Meier curve demonstrating the effect of hormone used as cytoreduction prior to HIFU (PSA </=0.5 ng/ml).

Figure 1 illustrates the PSA changes that occurred over the period of follow-up with mean PSA levels at baseline, 3, 6, 9, 12, 18, and 24 months of 8.3 ng ml^−1^ (range 7.01–9.41), 0.33 (range 0.22–0.45), 0.49 (range 0.28–0.69), 0.54 (range 0.26–0.82), 0.65 (range 0.27–1.02), 0.68 (range 0.39–0.97) and 0.57 (range 0.12–1.02). The proportion achieving PSA levels ⩽0.2 ng ml^−1^ for those who completed 3, 6, 9, 12, 18, and 24 months was 70, 65, 58, 58, 57, and 60.9%, respectively (Table 3) (Figure 2). We found no significant relationship between Gleason score, preoperative PSA, or T-stage and the ability to render a PSA nadir ⩽0.2 ng ml^−1^ (univariate analysis). In comparison 83, 78, 81, 78, 75, and 83% of men achieved ⩽0.5 ng ml^−1^, at 3, 6, 9, 12, 18, and 24 months, respectively (Figure 3). We analysed the effect of hormonal use on the ability to achieve a PSA nadir <0.2 and <0.5 ng ml^−1^ with the use of Kaplan–Meier curves. There was no statistically significant difference between the group on hormones and those without (Breslow (Generalised Wilcoxon) *P*=0.693 for PSA 0.2 ng ml^−1^ and *P*=0.146 for PSA 0.5 ng ml^−1^) (Figures 4 and 5). Of those achieving unrecordable PSA levels at 3 months follow-up, 23 out of 64 had hormones and 41 out of 64 had no hormones.

**Figure 6:**
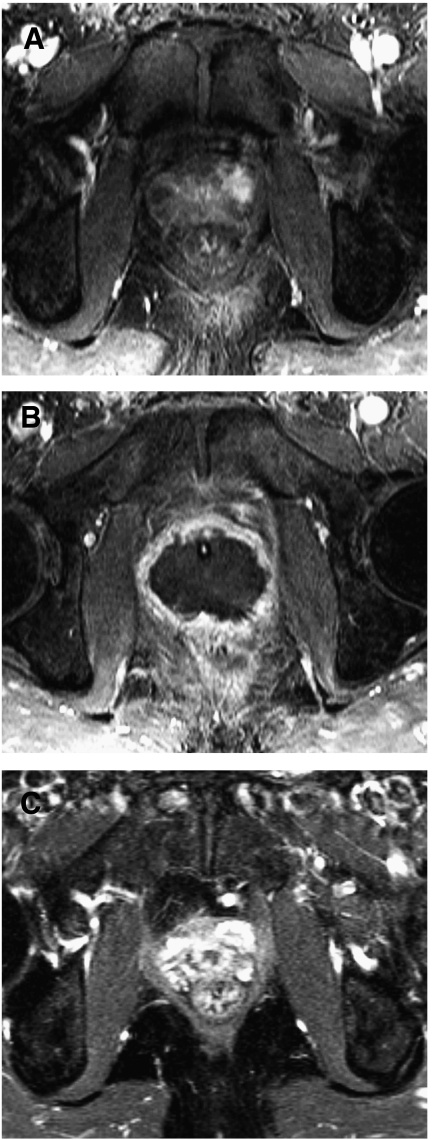
Contrast enhanced MRI changes in a successful treatment for prostate cancer using HIFU. (**A**) 1.5 Tesla dynamic contrast enhanced MRI using gadolinium prior to HIFU treatment demonstrating localised disease with a lesion in the left antero-lateral side of the gland (circled). (**B**) 1.5 Tesla dynamic contrast enhanced MRI using gadolinium at 2 weeks demonstrating poor perfusion in the prostate after HIFU treatment. Urethral catheter seen in-situ. (**C**) 1.5 Tesla dynamic contrast enhanced MRI using gadolinium at 6 months no residual prostate tissue and fibrotic reaction.

**Figure 7:**
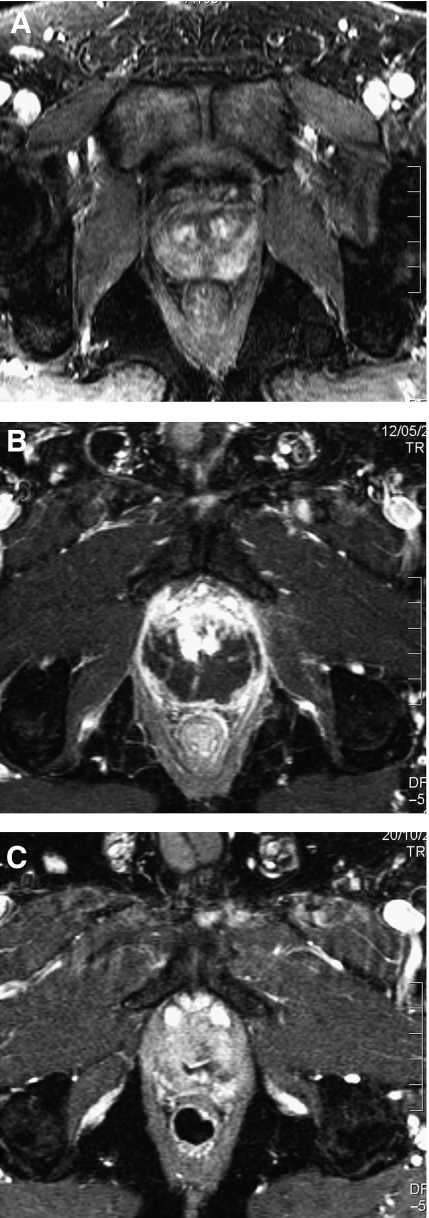
Contrast enhanced MRI changes in an under-treated prostate following HIFU. (**A**) 1.5 Tesla dynamic contrast enhanced MRI using gadolinium prior to HIFU treatment demonstrating localised disease with a lesion in the left postero-lateral side of the gland (circled). (**B**) 1.5 Tesla dynamic contrast enhanced MRI using gadolinium at 2 weeks demonstrating poor perfusion in the posterior prostate after HIFU treatment but an area of residual tissue with enhancement anteriorly (circled). (**C**) 1.5 Tesla dynamic contrast enhanced MRI using gadolinium at 6 months showing residual prostate tissue (circled).

Not all men accepted post-HIFU biopsies, because those in whom the PSA was >0.5 ng ml^−1^ but stable did not wish to have biopsies. Therefore, 31 patients were biopsied. 13 had proven histological evidence of either residual or recurrent cancer. The histology demonstrated Gleason score 7 in 5 out of 13 and Gleason score 6 in 7 out of 13 (ungradeable in 1 out of 13). Of the 13 with histological evidence of recurrent/residual cancer, eight opted for re-treatment with HIFU. Of these, three out of eight had biochemical control at 6–12 months (PSA <0.2 ng ml^−1^ in two out of eight and <0.5 ng ml^−1^ in one out of eight), one out of eight failed again (positive on biopsy) and underwent salvage external beam radiotherapy and four out of eight had insufficient follow-up to make meaningful comments about PSA kinetics (<6 months). The remainder who had positive biopsies (5 out of 13) opted for active surveillance of low volume, Gleason 3+3 residual prostate cancer. Figure 6A–C show a series of MRI scans demonstrating the ablation that is possible in a successful HIFU treatment whereas Figure 7A–C demonstrate incomplete ablation.

## Discussion

The natural history of prostate cancer prevents the use of mortality as an outcome measure in most short- to medium-term reports of prostate cancer therapy, so surrogates in the form of biochemical failure have emerged. However, the optimal definition of biochemical failure is far from clear. Indeed, owing to this lack of certainty the reporting of minimally invasive modalities has shown little consistency. The variability in biochemical outcome is demonstrated by the differing PSA nadirs used to define a successful outcome after cryosurgery or HIFU, with groups using any one of PSA ⩽1, ⩽0.5, ⩽0.4, ⩽0.3, ⩽0.2, and ⩽0.1 ng ml^−1^. PSA nadir ⩽0.2 ng ml^−1^ has evidence within radical prostatectomy series demonstrating its ability to predict a long-term outcome. Evidence for this particular level is currently insufficient for HIFU (Ganzer *et al*, 2008). A number of series use three successive PSA rises to define treatment failure according to the previous American Society for Therapeutic Radiology and Oncology (ASTRO) criteria to define biochemical failure after radiotherapy (ASTRO, 1997). This has its own drawbacks because the ASTRO criteria are not appropriate for evaluating PSA elevations sooner than 3 years follow-up and were validated for radiotherapy. Indeed, with the emergence of the ASTRO Phoenix criteria (nadir+2 ng ml^−1^) (Roach *et al*, 2006), the old definition is in itself questionable.

We have therefore chosen to report outcomes for three PSA values. At present, none of these parameters have been validated for HIFU or cryosurgery and for this reason we argue for their inclusion. The unrecordable level allows some comparison to radical prostatectomy in which this is achievable in the majority. PSA ⩽0.2 ng ml^−1^ has some evidence to demonstrate that it may be predictive of long-term cancer control following surgery and possibly after HIFU (Cookson *et al*, 2007; Ganzer *et al*, 2008). PSA ⩽0.5 ng ml^−1^ is an extra outcome measure that many ablative therapy series use so its inclusion may be of pragmatic use to allow comparison. Our early results demonstrate that the biochemical outcome in the first 2 years is seen uniformly throughout all three risk categories, although we accept that with greater follow-up cancer control is likely to deteriorate in the high-risk groups. A significant number in this series achieve early biochemical outcomes equivalent to radical prostatectomy (more than one-third achieve unrecordable PSA levels; 60% achieve ⩽0.2 ng ml^−1^). Furthermore, approximately 80% treated in this manner were able to achieve a PSA nadir of ⩽0.5 ng ml^−1^ within 3 months with this percentage maintained up to 12 months.

The evidence base for HIFU is growing with medium and long-term results now being reported. A total of 10 series have been published to date. These include medium-term results (10–30 months follow-up) (Gelet *et al*, 2001; Chaussy and Thuroff, 2003; Thuroff *et al*, 2003; Vallancien *et al*, 2004; Ficarra *et al*, 2006; Uchida *et al*, 2006a, 2006b; Poissonnier *et al*, 2007) and longer term results (5 years or more) (Blana *et al*, 2004, 2008). Two have used the Sonablate 500 and the remainder the Ablatherm. These series include HIFU re-treatments within the overall outcome data with mean HIFU sessions ranging from 1.17 to 1.4. In summary, incontinence rates ranged from 0.5 to 15.4%, impotence 13–53% and recto-urethral fistulae 0–2%. Biochemical control rates varied from 66 to 84% (ASTRO criteria) at mean follow-up that varied between 3 and 5 years. It is therefore encouraging that longer term data are now emerging from HIFU cohort series. The distinction between which series used which device is only of minor importance.

We have demonstrated that a cohort of men comprising a wide spectrum of risks can be treated with HIFU in a one-off, day-case setting in a safe manner with good biochemical outcomes. Although higher risk groups have been treated, it is important to note that with limited follow-up, the poorer outcomes seen in high-risk categories is likely to not have manifest itself. Nonetheless, impotence is still significant. To reduce morbidity further, focal therapy has been proposed as a paradigm shift in the management of prostate cancer. HIFU could fulfil this role as it is able to discretely ablate defined areas of tissue within the prostate (Ahmed *et al*, 2007). Focal therapy trials are currently ongoing at our centre and in a number of centres in the United States (Ahmed and Emberton, 2007).

Before addressing the policy implications of these findings certain important methodological considerations need to be addressed. First, there is incomplete reporting of outcome. For instance, whether men were evaluated by a questionnaire depended on which site they were treated. We cannot be sure that the same result would have arisen had all been tested. To assist the reader we have tried to document both the numerator and denominator so that the sample on which the variable is being evaluated is made explicit.

Second, our evaluation of genito-urinary outcomes was limited. Question 2 of the IIEF-15 does not assess whether there has been any change in the quality of the erections. This is an acknowledged clinimetric challenge (Burnett *et al*, 2007). We have attempted to mitigate this by reporting total IIEF-15 scores for all men who completed it at follow-up and using two thresholds for defining potency on question 2.

Third, the early post-treatment toxicity deserves comment, as it remains poorly understood. The rates we report are high and challenge the claim of the treatment being both minimally invasive and well tolerated. Infection and dysuria have been grouped together. Many were assumed by us and primary care physicians to be urinary tract infections and were treated as such, without evidence of a positive urine culture. Patients are now told that they should expect these symptoms. Having said this, the very high rate of epididymo-orchitis was certainly of bacterial origin. Now that suprapubic catheters are used combined with early removal, there may be a reduction in this complication. The issue of retained debris or stricture also deserves comment. If rates similar to those reported in this paper had persisted we would not have wanted to continue with this treatment. Most of the events were clustered to the early part of the series at the beginning of our learning curve and our threshold for intervention was very low. Any report of difficulty in voiding led to cystoscopy. At present, we intervene rarely and instruct the patients to self-catheterise if voiding is impaired, with an inability to catheterise resulting in cystoscopy. It is felt that self-catheterisation may aid in dislodging retained debris causing an acute deterioration in urine flow. Whether such a policy will result in fewer interventions, especially bladder neck incision and dilatation for stricture, is yet to be proven. Therefore, our early results clearly stand and it will be incumbent on subsequent analyses to establish the true rate of these side-effects in the most recent patients where our threshold for intervention has changed.

Fourth, the use of hormones for cytoreduction will have resulted in potential confounding. In all cases this was in the form of an antiandrogen with or without a 5 α-reductase inhibitor for 3 months. These were stopped on the day of the procedure. Dutasteride and finasteride have a half-life of 5–6 weeks and 5–8 h, respectively. Bicalutamide has a half-life of 6 days. We therefore argue that the influence of these hormonal agents would have been minimal by 3 months and virtually absent by 6 months. This assumption is supported by Kaplan–Meier curves which demonstrate no statistically significant difference between those taking hormones and those without (Figures 4 and 5).

Fifth, given the absence of formal exclusion/inclusion criteria it was inevitable that some key baseline data would not be available for analysis. Again, to mitigate this, we have not sought to exclude the latter group and have reported outcomes for all men. There was no difference in biochemical or histological outcome between those with full data that permitted D’amico risk stratification and those in whom it was not possible.

Sixth, the absence of systematic evaluation probably has the greatest impact on the way in which the status of presence or absence of disease was elicited from the treated prostate. In other words, it was only those in whom the PSA rose above 0.5 ng ml^−1^ or those in whom a rising PSA was seen were advised to have a biopsy. Only those who agreed to have this done could be evaluated. The inclusion of standard biopsies may have explained why the 3 months median PSA rises from 0.33 ng ml^−1^ at 3 months to 0.57 at 24 months. It is difficult to explain this. One could speculate that in a similar vain to prostate brachytherapy in which a ‘PSA bounce’ occurs between 1 and 3 years and can be as high as 2 ng ml^−1^ although the mean bounce is around 0.5 ng ml^−1^. The mechanism of tissue ablation is very different to brachytherapy so it is not immediately clear if and why such a bounce should occur. The increase may also represent an early sign of failure. However, with smaller numbers at 24 months follow-up it is difficult to ascertain whether this increase in PSA is a sustained one. Within a trial setting for a novel therapy the requirement for patients to undergo a protocol biopsy would be the standard. However, whole-gland HIFU used in the way described in this paper is not new and it was approved in the United Kingdom as a therapeutic alternative with the requirement for close follow-up by NICE. Therefore, the elicitation of the histological status was conditional on absolute PSA values or PSA kinetics. The bias that might result from this approach is an underestimation of residual or recurrent prostate cancer.

## Conclusions

HIFU in the treatment of prostate cancer can result in acceptable short-term levels of cancer control. Moreover, it can be used with a high degree of certainty that the man will remain continent afterwards and two-thirds might expect to have erections sufficient for penetration 1 year following treatment. Importantly for some, this can be achieved in a one-off treatment in an ambulatory setting with the majority discharged home the same day after a few hours. Nonetheless, our data remains immature and the determinants of outcome are not yet known. Clearly, longer term follow-up is required, but in a more systematic and prospective manner. The difficulty in recruiting to randomised controlled studies when comparing treatments for prostate cancer has been demonstrated with the latest cryosurgery *vs* radiotherapy randomised controlled trial that failed to recruit (Donnelly *et al*, 2007). As in other areas of emerging technology the most efficient and effective way to collect long-term prospective data may be through an online registry (McCulloch *et al*, 2002; Grilli and Taroni, 2006). Although, the recent NICE guidelines in the United Kingdom (Graham *et al*, 2008) have recommended HIFU as well as cryosurgery be used only if patient data are added prospectively into clinical trials, their implementation guidelines have clarified that national or international registries are also acceptable (NICE Implementation advice on Prostate Cancer Guidelines, 2008). The British Association of Urological Surgeons have followed suit on this advice and have opened their cancer registry to all cryosurgery and HIFU treatments for prostate cancer. There are industry-sponsored international registries for HIFU and cryosurgery already set up (Jones *et al*, 2008). Such registries must ensure impartiality with strict guidelines and data monitoring to ensure the data is a true representation of real practise. This should ensure that the lack of comparative data does not inhibit the timely diffusion of potentially successful technologies into mainstream clinical practise for the benefit of men undergoing treatment for prostate cancer.

## Figures and Tables

**Table 1:** Baseline demographic data for localised Prostate Cancer treated with HIFU

Number	172	
Age, years (median, s.d., range)	64.1 (s.d. 8.3) (47–88)	
Hormones^a^	49 (28.5%)	
		
	**No.**	**%**
*Pre-HIFU PSA (pre-cytoreduction) (ng ml*^−*1*^)		
0–4.0	24	15.9
4.1–10.0	85	56.3
10.1–20.0	37	24.5
>20.0	5	3.3
Unavailable	21	12.2
		
*Gleason*		
⩽6	89	54.6
=7	64	39.3
>7	10	6.1
Unavailable	9	5.2
		
*Stage*		
T1c	48	33.1
T2a	25	17.2
T2b	29	20.0
T2c	17	11.7
T3a	23	15.9
T3b	3	2.1
Unavailable	27	15.7
		
*Risk categories* b		
Low	38	27.9
Intermediate	51	37.5
High	47	34.6
Unavailable	36	20.9

a All cytoreduced with anti-androgen and 5-*α* reductase inhibitor for 3 months.

b D'amico risk categories were defined according to the 1998 criteria as follows:

Low: PSA ⩽10 ng ml^−1^ and Gleason ⩽6 and Stage ⩽T2a (For low risk, all these parameters must be met); Intermediate: PSA 10.1–20 ng ml^−1^ or Gleason ⩽7 or Stage T2b (For intermediate, any one of these or a combination of all of these equals intermediate, provided no high-risk parameters are present); High: PSA >20 ng ml^−1^ or Gleason 8–10 or Stage ⩾T2c (For high risk, the presence of any of these parameters equals high risk).

**Table 2:** Operative factors and morbidity outcome for localised prostate cancer treated with HIFU

General Anaesthesia				172 (100%)	
Mean follow-up (days) (s.d., range)				346 (237, 135–759)	
*Catheter type*					
Urethral				58.4% (94)	
Suprapubic				41.6% (67)	
Not known				6.4% (11)	
Catheterisation time, days (mean, s.d.)				13.9 (8.3)	
*No. of patients requiring intervention for stricture/necrotic tissue*				30.2% (52)	
Dilatation for stricture (local anaesthesia)				15.7% (27)	
Intervention for necrotic tissue/stricture (general anaesthesia) (eight requiring two or more procedures)				18.0% (31)	
Bladder neck incision for stricture (one requiring two procedures)				10.5% (18)	
*Intervention for stricture/necrotic tissue by catheter type*					
Urethral				40.4% (38 out of 94)	
Suprapubic				19.4% (13 out of 67) * (Pearson χ*^*2*^, *P*=*0.005*)	
Urinary tract infection/dysuria				23.8% (41)	
Epididymitis				7.6% (13)	
Stress urinary incontinence (Grade 1) (no pads)				7.6% (13)	
Stress urinary incontinence (Grade 3)				0.6% (1)	
Recto-urethral fistulae				0% (0)	
					
*Potency*					
Using cohort of men who were potent pre-operatively on questionnaire and had filled in questionnaires at 3, 6, 9, and 12 months. Defined as scoring either 2 or 3 and above on IIEF-15 question 2 ‘When you had erections with sexual stimulation, how often were your erections hard enough for penetration?’					
					
**Months**	**0**	**3**	**6**	**9**	**12**
*N* (Score ⩾2)	24/24	9/24	11/24	14/24	16/24
% (Score ⩾2)	100	37.5	45.8	58.3	66.7
*N* (Score ⩾3)	12/12	5/12	7/12	8/12	8/12
% (Score ⩾3)	100	41.7	58.3	66.7	66.7
*N*	94	92	77	51	34
IIEF-15 Score	33.8	18.1	39.1	23.9	28.1
s.d.	(22.16)	(14.67)	(22.09)	(19.51)	(21.46)
*T*-test (comparison with baseline (*P*-value))	NA	<0.01	0.12	0.06	0.19
Mean IPSS Score	6.7	12.0	9.2	7.9	7.8
s.d.	5.84	8.60	8.00	7.51	7.33
*T*-test (comparison with baseline (*P*-value))	NA	<0.01	0.02	0.32	0.42

**Table 3:** Biochemical and biopsy outcome for localised prostate cancer treated with HIFU

**Follow-up (months)**	**3**	**6**	**9**	**12**	**18**	**24**
*N*	155	120	103	83	63	23
						
*Biochemical parameters*						
*PSA (ng ml*^−*1*^)						
<*0.05*						
*n*	64	43	36	29	26	10
**%**	41.2	35.8	35.0	35.0	41.3	43.5
*⩽0.2*						
*n*	108	78	60	48	36	14
**%**	69.7	65.0	58.3	57.8	57.1	60.9
*⩽0.5*						
*n*	129	94	83	65	47	19
**%**	83.2	78.3	80.6	78.3	74.6	82.6
